# The Application of Nanopore Sequencing Technology to the Study of Dinoflagellates: A Proof of Concept Study for Rapid Sequence-Based Discrimination of Potentially Harmful Algae

**DOI:** 10.3389/fmicb.2020.00844

**Published:** 2020-05-08

**Authors:** Robert G. Hatfield, Frederico M. Batista, Timothy P. Bean, Vera G. Fonseca, Andres Santos, Andrew D. Turner, Adam Lewis, Karl J. Dean, Jaime Martinez-Urtaza

**Affiliations:** ^1^Centre for Environment, Fisheries and Aquaculture Science, Dorset, United Kingdom; ^2^Roslin Institute, Edinburgh, United Kingdom; ^3^Scientific and Technological Bioresource Nucleus (BIOREN), Universidad de La Frontera, Temuco, Chile

**Keywords:** nanopore sequencing, harmful algal bloom, dinoflagellate, MinION, sequencing, alexandrium, eDNA

## Abstract

Harmful algal blooms (HABs) are a naturally occurring global phenomena that have the potential to impact fisheries, leisure and ecosystems, as well as posing a significant hazard to animal and human health. There is significant interest in the development and application of methodologies to study all aspects of the causative organisms and toxins associated with these events. This paper reports the first application of nanopore sequencing technology for the detection of eukaryotic harmful algal bloom organisms. The MinION sequencing platform from Oxford Nanopore technologies provides long read sequencing capabilities in a compact, low cost, and portable format. In this study we used the MinION to sequence long-range PCR amplicons from multiple dinoflagellate species with a focus on the genus *Alexandrium*. Primers applicable to a wide range of dinoflagellates were selected, meaning that although the study was primarily focused on *Alexandrium* the applicability to three additional genera of toxic algae, namely; *Gonyaulax*, *Prorocentrum*, and *Lingulodinium* was also demonstrated. The amplicon generated here spanned approximately 3 kb of the rDNA cassette, including most of the 18S, the complete ITS1, 5.8S, ITS2 and regions D1 and D2 of the 28S. The inclusion of barcode genes as well as highly conserved regions resulted in identification of organisms to the species level. The analysis of reference cultures resulted in over 99% of all sequences being attributed to the correct species with an average identity above 95% from a reference list of over 200 species (see Supplementary Material 1). The use of mock community analysis within environmental samples highlighted that complex matrices did not prevent the ability to distinguish between phylogenetically similar species. Successful identification of causative organisms in environmental samples during natural toxic events further highlighted the potential of the assay. This study proves the suitability of nanopore sequencing technology for taxonomic identification of harmful algal bloom organisms and acquisition of data relevant to the World Health Organisations “one health” approach to marine monitoring.

## Introduction

Aquatic microalgae fix carbon, release oxygen and provide a source of food for grazing organisms, and as such are essential components of the trophic web supporting healthy freshwater and marine environments. However, under certain conditions, the proliferation of these algae can have detrimental effects on the surrounding environment, commonly referred to as harmful algal blooms (HABs) (Hallegraeff et al., 2004). Although a natural phenomenon, HABs can be exacerbated and/or caused by anthropogenic activities such as, but not limited to, shipping, eutrophication and global warming (Hallegraeff and Bolch, 1991; Burkholder, 1998; Glibert et al., 2005; Bolch and de Salas, 2007; Estrada et al., 2008; Moore et al., 2009; Hallegraeff, 2010; Tatters et al., 2013; Glibert, 2017; Anderson et al., 2019). Furthermore, as global human population increases so do these influences on the environment, and as such there has been an increase in HAB events and their severity in recent years (Hallegraeff, 1993, 2010; Glibert, 2017; Anderson et al., 2019). Many species of microalgae, including those that form blooms, have the potential to produce a range of toxins. These toxins bioaccumulate in bivalve shellfish, which if consumed pose a threat to human health (Bauder et al., 2001; Kwong et al., 2006). These toxins are commonly categorized by their symptomatic manifestations and include, Paralytic, Amnesic and Diarrhetic shellfish poisons or PSP, ASP and DSP respectively (Hallegraeff et al., 2004).

The toxic algal species associated with HAB events belong to a variety of planktonic taxa. However, there is a notable dominance by protists of the Phylum *Dinoflagellata* in marine HAB events (Hallegraeff, 2004; Hernández-Becerril et al., 2007; Hallegraeff, 2010; Ralston et al., 2011; Turner et al., 2018). Of greatest concern are the acute and potentially fatal effects of PSP, a syndrome associated with consumption of saxitoxin (STX), which causes paralysis and can result in death from suffocation. The production of STX in temperate marine environments is primarily associated with the genus *Alexandrium* but has also been linked to *Gymnodynium* and *Pyrodinium* in tropical and subtropical regions (Ichimi et al., 2002; Etheridge, 2010; Wiese et al., 2010; Terrazas et al., 2017).

Routine monitoring of water samples for the causative organisms of HABs is usually fulfilled by fixing water samples with Lugol’s solution and manually observing samples in Utermöhl chambers under a light microscope (LM), a technique that has seen little development in decades (Utermöhl, 1931). The robustness of this method is well proven and there is limited motivation to modernize the technique. However, analysis by LM is unable to distinguish between toxic and non-toxic species with similar morphology such as those within the genus *Alexandrium*, or identify small organisms such as *Azadinium* spp. Furthermore, these analyses are time consuming, and require highly skilled personnel. The need to address such limitations has resulted in development of alternative techniques. These include the use of flow cytometry or molecular tools. Examples include: sandwich hybridization assays (SHA), fluorescence *in situ* hybridization (FISH), microarrays, quantitative polymerase chain reaction (qPCR) and real time PCR (RT-PCR) (Buskey and Hyatt, 2006; Bott et al., 2010; Kudela et al., 2010; Poulton and Martin, 2010; Zamor et al., 2012; Rees et al., 2014; Medlin and Orozco, 2017). Although these technologies are applied widely in research and localized monitoring, their high specificity means that application to widespread routine monitoring would require innumerable parallel or multiplexed assays to be performed on each sample (Bott et al., 2010), often rendering them uneconomical or impractical. At the time of publication of this manuscript only one laboratory has achieved ISO 17025 accreditation for any of these techniques.

An alternative technology, proven to give greater taxonomic coverage is the massively parallel sequencing of amplicons generated from environmental DNA samples, commonly referred to as eDNA metabarcoding (Valentini et al., 2016). This approach has the potential to give information on a broad diversity of organisms within a tested sample, depending on the primers used, and has been widely used for research purposes since the advent of high throughput sequencing (HTS), formally referred to as Next Generation Sequencing (NGS) (Shendure and Ji, 2008; Bott et al., 2010; Tan et al., 2015). HTS instruments are bulky and expensive, limiting its application to centralized laboratories and research projects (Batovska et al., 2017).

The MinION, manufactured by Oxford Nanopore Technologies (ONT), provides an attractive alternative to HTS sequencing. Nanopore sequencing can process exceptionally long nucleic acid molecules, including both RNA and DNA. The resulting reads routinely exceed 20 kb in length (Laver et al., 2015; Schalamun et al., 2019). The MinION, with its low cost and portability, has the potential to revolutionize laboratory and field detection of HABs as it has started to for pathogen detection and environmental analysis (Kilianski et al., 2015; Marx, 2015; Quick et al., 2016).

A perceived disadvantage associated with nanopore sequencing is the high error rate when compared to other platforms. These errors tend to be associated with homopolymeric regions and manifest as insertions or deletions (indels) in the sequence (Rang et al., 2018). To overcome this limitation, sympathetic interpretation strategies are adopted, for example, percent identity rather than percent accuracy is used to compare sequences. Identity provides a measure of similarity without taking into account sequence length or gaps (May, 2004) and as such, indels have no, or lower, negative impact on alignment accuracy. The use of identity is therefore a powerful and simple method to analyze raw nanopore reads, prior to bioinformatic manipulation.

High-throughput nanopore sequencing often requires the use of multiple specialist bioinformatic tools to manipulate and analyze very large datasets. This usually requires highly specialized programming techniques, an obstacle which is often viewed as a disadvantage. However, these methods are essential as they facilitate production of highly accurate data from nanopore derived sequences. This is achieved by aligning multiple reads (between 10 and > 10^6^) from a single source and generating a consensus sequence. The generation of consensus sequences also provides information on variations within a dataset referred to as single nucleotide polymorphisms (SNP) (Koren et al., 2017; Shabardina et al., 2019). Information on SNP data within a genome can be valuable when studying genetically similar species, sub species or even individuals (Rafalski, 2002).

The aim of this study was to explore the suitability of the MinION platform for the detection and discrimination of dinoflagellates in environmental samples. By focusing method development on the genus *Alexandrium* due to its association with PSP and challenges in taxonomic discrimination between toxic and non-toxic variants, the study highlights the specificity of the assay. Due to the diversity of dinoflagellate HAB taxa, and their importance to environmental health, additional genera were included in the validation, namely *Gonyaulax, Prorocentrum*, and *Lingulodinium*. Furthermore, approximately 100 genera were included in the data analysis tool and a customized PCR method was adopted suitable for the large and highly complex dinoflagellates genomes (Benítez-Páez and Sanz, 2017; Casabianca et al., 2017). The PCR primers selected for this study amplified a 3 kbp region, encompassing a large proportion of the rDNA cassette. The ability of the MinION to sequence this relatively large amplicon meant that multiple barcoding regions could be included (Walsh et al., 1998; John et al., 2003; Litaker et al., 2007; Orr et al., 2011; Stern et al., 2012; Wang et al., 2014). This combined with the systems portability are key features unique to this technology.

This study strived to use web based, user friendly data analysis options, namely EPI2ME (ONT/Metrichore, Ltd.) and NanoPipe (University Muenster)^1^ in addition to specialist bioinformatics software pipelines. In doing so, providing user friendly “online” options and custom data analysis pipelines available “off line” that require greater expertise and local computing power.

The performance of the assay was assessed by the analysis of known control morphospecies and mock community analysis in representative environmental sample matrices, and the analysis of HAB event samples. In addition, Sanger sequencing was undertaken and used as a comparative gold standard to assess sequencing performance of the MinION.

By Taking Advantage of the Portability, Low Cost and User-Friendly Nature of the Minion Platform, This Study Assesses This Exciting Technology and Its Application to the Study of Harmful Marine Algae. the Benefits and Limitations of Minion Application in This Context Are Subsequently Discussed.

## Materials and Methods

### Reference Culture Acquisition and DNA Extraction

Environmental water samples were collected in 1L sterilized flasks, immediately fixed using Lugol’s solution and chilled as soon as possible after collection. Once returned to the laboratory they were kept at 4°C until DNA extraction could be undertaken (within 1 week of collection for sites 1–3 and 1 month for sites 4 and 5).

The following cultures were acquired from Culture Collection of Algae and Protozoa (CCAP, Oban, Scotland), Marine Biological Association (MBA), Plymouth, UK and Culture collection of Marine Protozoa (CCMP), Bigelow, Maine, United States.: *Alexandrium tamarense* (CCAP1119/31), *Alexandrium tamutum* (CCAP1119/51), *Alexandrium minutum* (MBA733), *Alexandrium catenella^∗^* (CCAP1119/52), *Gonyaulax spinifera* (CCAP1118/2) and *Lingulodinium polyedrum* (CCAP1121/2), and *Alexandrium fundyense* (CCMP1719). *^∗^Alexandrium catenella* and *Alexandrium fundyense* are accepted as the same species and although *catenella* is now the accepted name the synonym *fundyense* is present on a substantial amount of online material, therefore in this paper *catenella* is used but *A*. *fundyense* appears where data for sequences were generated under this name, making identification of source material straight forward.

Cultures were grown at 17°C in 50 mL (25 cm^2^ growth area flask) flasks using L1 media. The culture was exposed to 14 h of light and 10 h of darkness per day and checked for the presence of live cells prior to fixation with Lugol’s solution, before DNA extraction (Higman and Turner, 2010).

Fifty milliliter aliquots of the fixed environmental water samples and varying volumes of algal cultures, dependant on cell density were centrifuged at 4500 g for 10 min. The supernatant was discarded, and the resulting pellet was used for DNA extraction. DNA extraction was carried out using the Qiagen Power Biofilm DNA isolation Kit (Qiagen, Hilden, Germany) with lysis facilitated using MPBio Fast prep system (MPbio, Solon, OH, United States) set to full speed for 30 s. DNA extracts were stored at −20°C until PCR amplification was undertaken (Hatfield et al., 2019).

### Long Range PCR Protocol

**FIGURE 1:**
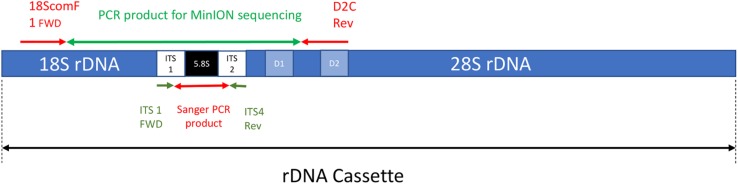
A simplified depiction of the rDNA cassette including the approximate location of both forward and reverse primers for the MinION sequencing and Sanger sequencing PCR reactions (note not to scale).

The forward primer 18ScomF1 (Zhang et al., 2005) and the reverse primer D2C (Scholin et al., 1993) were used to amplify a fragment with approximately 3020 bp comprising the almost complete sequence of the small subunit ribosomal RNA gene (18S), internal transcribed spacer 1 (ITS1), 5.8S ribosomal RNA gene (5.8S), internal transcribed spacer 2 (ITS2), and up to the D2 region of the large subunit ribosomal RNA gene (28S) (Figure 1). These primers were tailed as recommended by ONT to allow barcoding using the ONT PCR barcode kit (EXP-PBC001) as shown below:

Tailed-18ScomF1
5′ **TTTCTGTTGGTGCTGATATTGC**GCTTGTCTCAAA GATTAAGCCATGC 3′

Tailed-D2C
5′ **ACTTGCCTGTCGCTCTATCTTC**CCTTGGTCCGTG TTTCAAGA 3′

The first 22 characters in bold and underlined are the tail sequences followed by 18ScomF1 and D2C primer sequences.

All PCR reactions were done in an Eppendorf Master Cycler Nexus, (Eppendorf, Hamburg, Germany). PCR reagents were sourced from New England Biolabs (NEB). The initial long-range PCR reaction was done in 50 μL volumes composed of: 10 μL of 5X Phusion HF buffer, 1 μL of dNTPs (10 mM), 2.5 μL of each primer (10 μM), 1 μL Phusion DNA polymerase, 28 μL of dH_2_O and 5 μL of DNA extract. The PCR was run using the following thermal regime: 98°C for 60 s, followed by 30 cycles of 98°C for 10 s, 63°C for 20 s, 72°C for 90 s and a final extension of 72°C for 10 min. Resulting amplicons were cleaned using Agencourt AMPure XP, and quantified by Qubit 3.0, using the 1X dsDNA high sensitivity kit (Thermo Fisher Scientific, cat#: Q33230), additional analysis by NanoDrop was performed to assess DNA purity.

### Sanger Sequencing Protocol

The forward primer ITS1 (5′ GGT GAA CCT GAG GAA GGA T 3′) and the reverse primer (5′ TCC TCC GCT TAT TGA TAT GC 3′) (Stern et al., 2012). were used to amplify a fragment comprising ITS1, 5.8S, and ITS2 with approximately 550 bp of each pure culture of *A. tamarense, A. tamutum, A. catenella, A. minutum, G. spinifera*, and *L. polyedrum*. PCR reaction was done in 50 μL volumes composed of: 10 μL of 5X Phusion HF buffer, 1 μL of dNTPs (10 mM), 2.5 μL of each primer (10 μM), 1 μL Phusion DNA polymerase, 28 μL of dH_2_O and 5 μL of DNA extract. PCR was performed using the following thermal regime: 94°C for 3 min, followed by 35 cycles of 95°C for 30 s, 47°C for 30 s, 72°C for 45 s and a final extension of 72°C for 7 min. PCR products were purified using HT ExoSAP-IT^TM^ High-Throughput PCR Product Cleanup (Applied Biosystems) as described by the manufacture. The purified PCR products were sent for direct Sanger sequencing in Eurofins Genomics (Ebersberg, Germany) using both the forward and reverse primers.

### Mock Community and Environmental Samples

A mock community was created by enumerating pure cultures of *A. tamarense, A. tamutum, A. catenella, and A. minutum* using a hemocytometer. From the calculated cell concentration, the volume required to transfer 100 ± 20 cells of each species was then determined. Example matrices were spiked with these volumes, from each culture, to establish the same mock community in each example matrix. In total during the project five environmental samples were sourced. Of these three were used as example matrices to be spiked with the mock community. The three example matrices chosen each came from different environment types and all within 5 km of each other, namely, a tidal lagoon (site 1), a rocky cove (site 2) and open water (approximately 2 km offshore, site 3). To generate a positive control for the mock community analysis cells were also spiked into sterilized sea water (sand filtered, UV treated and sterilized by autoclaving at 121°C for 15 min). Finally, sterilized sea water with no cell addition was used as a negative control. The presence of pre-existing HAB organisms in the environmental samples used for the mock community analysis was checked at Site 1, as this site was located on shellfish harvesting area which at the time of collection had no HAB cells present or toxins detected in shellfish samples as determined by official control monitoring conducted at the site.

The remaining two environmental samples, Site 4 and Site 5, were not used for the mock community analysis as they were specifically collected from locations experiencing low level toxic events. Site 4 had been closed due to a DSP event with *Dinophysis* counts of 200 cells/L and Okadaic Acid levels of 81 μg/Kg in shellfish. Site 5 was experiencing low, sub-closure PSP event with *Alexandrium* counts of 160 cells/L and saxitoxin levels of 42μg STXeq/KG.

### Method Reproducibility

To ascertain the minimum number of reads assigned to a species required to provide an accurate representation of the sample a co-efficient of variation was assessed, calculating relative standard deviations (RSD) for each species. This was done by preparing a single sample into four barcodes and sequencing them concurrently on the same instrument. The relative standard deviation of alignments for each species was calculated from the four barcodes and plotted against the average number of alignments for each respective species.

To assess inter-batch PCR reproducibility, two PCR reactions were prepared using DNA extract from site 4. The two runs were barcoded and run sequentially to eliminate instrumental variability. A Chi-squared test was applied to the 100 most abundant species in the resulting two data sets to ascertain if they were significantly different.

Instrumental inter-batch variability was assessed by running a single sample from Site 5 in parallel on two different instruments. Again, a Chi-squared test was applied to the 100 most abundant species in the resulting two data sets to ascertain if they were significantly different.

A further *in silco* investigation into the amount of data required to be representative was undertaken. This was achieved by uploading seven geometrically smaller datasets (factor of two), from approximately 2,500–200,000 reads for site 1 environmental sample.

### Sample Preparation and MinION Sequencing

The ONT protocol for “PCR barcoding amplicons (SQK-LSK109)” was performed following manufacturers’ instructions. Briefly, amplicons from the first PCR reaction obtained using the tailed primers were purified using AMPure XP beads (AmbipureX) and quantified using Qubit and NanoDrop. Each sample was diluted to 100–200 fmol and mixed with 2 μL of the respective barcode (barcodes BC01 to BC12), 50 μL of LongAMP Taq 2X mastermix and made up to a total volume of 100 μL with Nuclease-free water. The PCR reaction was then done using the following thermal conditions: 95°C for 2 min, followed by 15 cycles of 95°C for 15 s, 62°C for 15 s, 65°C for 90 s and a final extension of 65°C for 10 min. The barcoded amplicons were then purified using AMPure XP beads, quantified using Qubit and pooled into a library containing a total of 1 μg of DNA. A DNA repair reaction using NEBNext FFPE DNA repair mix (M6630) was carried out by incubation at 20°C for 5 min followed by AMPure XP beads clean-up, and ligation using NEBNext Quick Ligation Module (E6056). This was followed by AMPure XP beads clean-up and quantification using Qubit. The library was combined with loading beads and sequencing buffer before being transferred to a MinION R9.4 flow cell. The MinION was run for 36 h using the MinIT device. Note: negative control samples were prepared by using average sample volumes for each step to be representative of sample preparation.

### Bioinformatic Analysis

Local base calling of Fast5 files was performed using the MinIT (ONT-minit-release 19.05.2) device with the “flipflop” algorithm. Demultiplexing was performed using Porechop (v0.2.3)^2^. Analysis of the resulting sequences was performed on the custom alignment tool on the EPI2ME platform via the ONT website provided by Metrichor (Cambridge, United Kingdom). This workflow makes use of minimap2 version 2.12 and reference database to determine average accuracy and identity. The reference database was created by selecting 18S sequences of marine species from the DinoRef database (Mordret et al., 2018) including 233 species of dinoflagellate from 99 genera. Additionally, sequences from two species of diatoms were added. The downloaded sequences were aligned using Clustaw multiple alignment tool on BioEdit software version 7.5.0.3. and trimmed resulting in sequences with 1.3–1.8 kbp in length. The resulting Fasta file was uploaded using EPI2ME desktop agent via the Fasta reference upload, workflow. Fastq files was uploaded using EPI2ME Desktop agent for demultiplexing and filtering (sequence quality > Q7). Reads were also filtered using a Q8 threshold to compare with Q7 filtered reads.

A custom data analysis pipeline was developed and used to assess the pure culture data. It involved filtering reads by length (>2800 bp) and quality (>10) using NanoFilt 1.1.0 (De Coster et al., 2018). Adapters and barcodes were trimmed with qcat 1.1.0^3^. Taxonomic assignment was carried out with the bioinformatic tool Centrifuge 10.3-beta (Kim et al., 2016), using a reference list consisting of 82 representative dinoflagellate sequences sourced from NCBI, taxonomic assignation was performed based on a threshold of 95% of identity configured by -min-totallen option of centrifuge bioinformatic tool. Plots and analysis of taxonomic abundance were made with Pavian 0.3^4^.

Consensus nucleotide sequences were created for *A. tamarense, A. tamutum, A. catenella, A. minutum, G. spinifera*, and *L. polyedrum* using Canu (v1.8), with default parameters for nanopore sequence data (Koren et al., 2017). Five thousand reads were randomly extracted for each species from the analysis of pure cultures and used to create the consensus. The quality of sequencing chromatograms from Sanger sequences was checked using Bioedit version 7.0.9.0. Sequence identity between *A. tamarense, A. tamutum, A. catenella, A. minutum, G. spinifera*, and *L. polyedrum* sequences available in GenBank and the Sanger sequences obtained in the present study was determined using BLASTn (Altschul et al., 1990). The consensus nucleotide sequences were also analyzed using BLASTn. Moreover, the sequence identity between the consensus and the Sanger sequences was performed using Bioedit.

To assess intragenomic polymorphism, NanoPipe was used to identify possible single nucleotide polymorphisms (SNPs) using default parameters (Shabardina et al., 2019). The consensus sequences were used as the target and the same 5000 reads used to create the consensus were used as the query.

### Chemical Analysis

Paralytic shellfish toxin (PST) analysis was performed using high performance liquid chromatography with fluorescence detection and pre column oxidation, in alignment with EU specifications (Turner et al., 2009, 2011; Hatfield et al., 2016). Lipophilic toxin detection was performed using ultra high-performance liquid chromatography with triple quadrupole mass spectroscopy (UHPLC-MS/MS) (European Union Reference Laboratory for Marine Biotoxins, 2015).

### qPCR Analysis

All environmental samples were screened for the presence of *Alexandrium* rDNA by qPCR (R.G. Hatfield et al., 2019). The forward primer Alex-FWD (5′ TGTTGCGGTTAAAAAGCTCGTAG 3′), reverse primer Alex-REV (5′ TGCACTTGACTGTGTGGTGTM 3′) and Alex MGB probe (5′ TGAGTATYTGGCACAGCC 3′) were used with the following thermal cycle: 37°C for 10 min, 95°C for 10 min and 50 repeat cycles of 95°C for 15 s and 63°C for 1 min. A standard curve was generated using a serial dilution ranging from 100,000 to 10 copies of purified DNA. Each sample was analyzed in triplicate and an average result used to estimate abundance.

## Results

### MinION Analysis of Cultures

**FIGURE 2:**
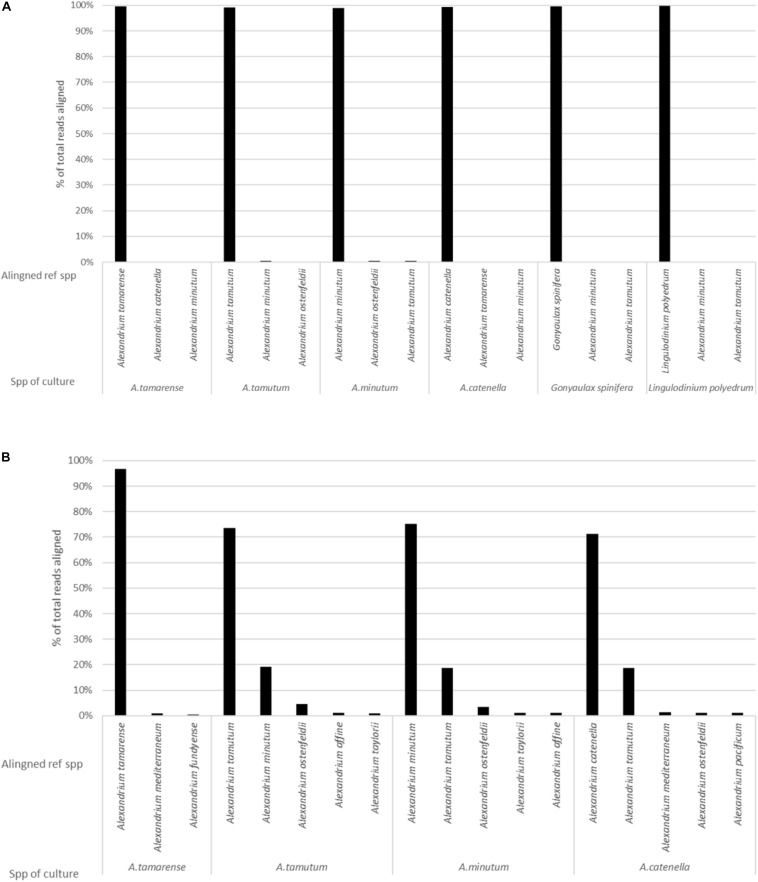
The results generated from analysis of pure cultures, showing the percentage of reads aligning to different spp. on the reference list for: **(A)** EPI2ME platform and **(B)** Custom data analysis pipeline.

The MinION was stopped after 36 h, generating over 5 million reads. It was however, identified that smaller volumes of data were enough to both generate consensus sequences (5000 reads for this data) and to produce representative data, as highlighted by Figure 4. As such a dataset consisting of 104,000 reads was used for the analysis. The data had an average Qscore of 9.21 and average length of 2,928 bases. Figure 2A shows the number of reads being attributed to the correct reference sequence as well as the 2 s most commonly assigned spp. with the Qscore threshold set to seven.

The results for the comparative assessment of Qscore seven and eight data thresholds are shown in Table 1. The higher Qscore threshold reduced the amount of data forwarded to analysis, from 96% for Qscore seven to 85% for Qscore eight. However, 2% more of the data passing Qscore eight threshold was successfully aligned to sequences in the reference list. The percentage of sequences for each pure culture aligning to the correct reference species was marginally higher for the Qscore eight threshold but only by 0.1%. Furthermore, the average percent identity improved by 0.4% for each of the samples.

**TABLE 1:** Data from pure culture analysis with comparison of Q score 7 and 8 filtered data.

Ref culture	Ref sequence aligned to (Q7/Q8)	Qscore 7 threshold				Qscore 8 threshold			
		# sequences aligned	Total	% of total	Average % ident	# sequences aligned	Total	% of total	Average % ident
*A. tamarense*	Alexandrium tamarense	7,047		99.4%	96.1%	6,136		99.5%	96.5%
	Alexandrium catenella	13	7088	0.2%	93.7%	6	6166	0.1%	95.4%
	Alexandrium minutum	5		0.1%	94.8%	3		0.0%	96.3%
*A. tamutum*	Alexandrium tamutum	12,410		99.1%	96.0%	10,724		99.3%	96.4%
	Alexandrium minutum	51	12523	0.4%	95.3%	34	10797	0.3%	96.2%
	Alexandrium ostenfeldii/tamarense	18		0.1%	94.4%	10		0.1%	96.5%
*A. minutum*	Alexandrium minutum	9,953		98.9%	96.1%	8,667		99.0%	96.5%
	Alexandrium ostenfeldii/tamutum	44	10068	0.4%	94.8%	34	8754	0.4%	95.7%
	Alexandrium tamutum/ostenfeldii	43		0.4%	95.1%	31		0.4%	95.8%
*A. catenella*	Alexandrium catenella	8,380		99.3%	95.1%	7,284		99.4%	95.5%
	Alexandrium tamarense	28	8441	0.3%	95.4%	22	7328	0.3%	95.8%
	Alexandrium minutum	7		0.1%	95.3%	5		0.1%	96.8%
*Gonyaulax*	Gonyaulax spinifera	9,656		99.5%	96.1%	8,420		99.6%	96.5%
*spinifera*	Alexandrium minutum	15	9704	0.2%	95.0%	12	8455	0.1%	95.4%
	Alexandrium tamutum	11		0.1%	94.1%	7		0.1%	94.5%
*Lingulodinium*	Lingulodinium polyedrum	11,202		99.7%	95.9%	9,688		99.7%	96.3%
*polyedrum*	Alexandrium minutum	12	11237	0.1%	96.7%	10	9715	0.1%	97.2%
	Alexandrium tamutum/tamarense	7		0.1%	93.9%	6		0.1%	94.5%

The custom data processing pipeline only included the *Alexandrium* spp. and returned the same overall probable identifications as the EPI2ME results. Figure 2B gives a simplified account of the dataset. *Alexandrium tamarense* had the highest correctly attributed sequences 97% of the alignments attributed correctly, the other cultures had lower values ranging between 70% for *Alexandrium catenella* and 75% for *Alexandrium minutum*. Almost all the wrongly attributed reads for *Alexandrium minutum* and *Alexandrium catenella* aligned with the *Alexandrium tamutum* reference sequence.

### Comparison of Sanger and Nanopore Sequences

Sanger sequences ranged in length from 389 and 580 bp, with individual sequence alignments between MinION and Sanger and a summary of the findings available in Supplementary Material 2. In all cases the alignments between MinION consensus and Sanger sequences had over 99% identity except for *A. cateonella*, which only achieved 96.18%. All discrepancies between Sanger and MinION sequences were attributable to indels in homopolymeric regions, again except for *A. catenella*, which had only one of 14 discrepancies attributable to this. The Sanger sequence for *A. catonella* was also the only sample not to achieve 100% identity with a reference sequence when BLAST was performed on the NCBI database (99.23% accession: KF646477.1). Conversely the MinION consensus sequence for *A. catonella* was the only one of two consensus sequences to achieve 100% identity, along with *G. spinifera* with references on NCBI database (KF646487.1 and FRR865625.1, respectively).

### Mock Community Analysis

**FIGURE 3:**
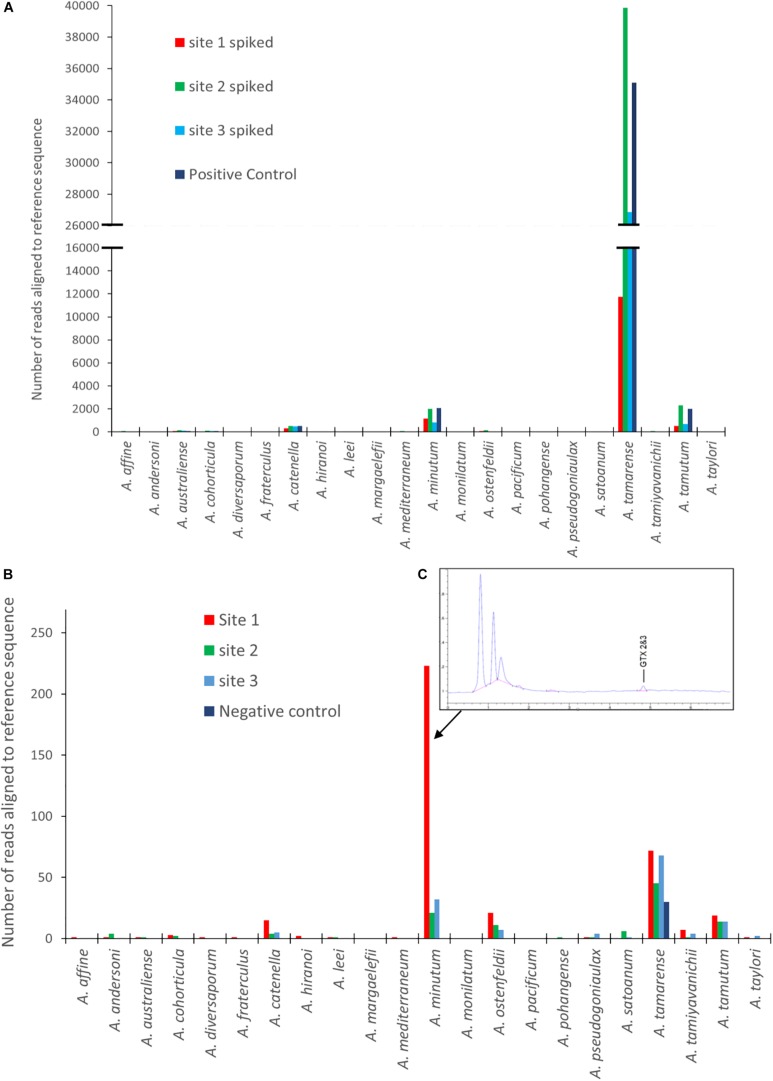
Results from EPI2ME alignment after MinION sequencing for: **(A)** Spiked environmental samples (due to the larger abundance of *A. tamarense* sequences the X axis to be split so as to see of lower copy number species. **(B)** Environmental samples from each site as well as a negative control. **(C)** An HPLC-Fld chromatogram showing saxitoxin profile from site 1. (Note: sequence alignments shorter than 1000 bp removed for both A and B analyses).

Figures 3A,B are graphical representations of the mock community data showing the number of sequences aligning to each of the *Alexandrium* species on the reference list. It includes the spiked and un-spiked samples for each site matrix as well as the control. There is a notable difference in the number of reads attributed to each of the four different species of *Alexandrium* which is reflected in each of the different matrices. *A. tamarense* had between approximately 40 and 75 times more sequences aligned depending on the matrix, than *A. catenella*, attributable to species specific variation in cellular copy number (Bott et al., 2010; Wick et al., 2018).

**FIGURE 4:**
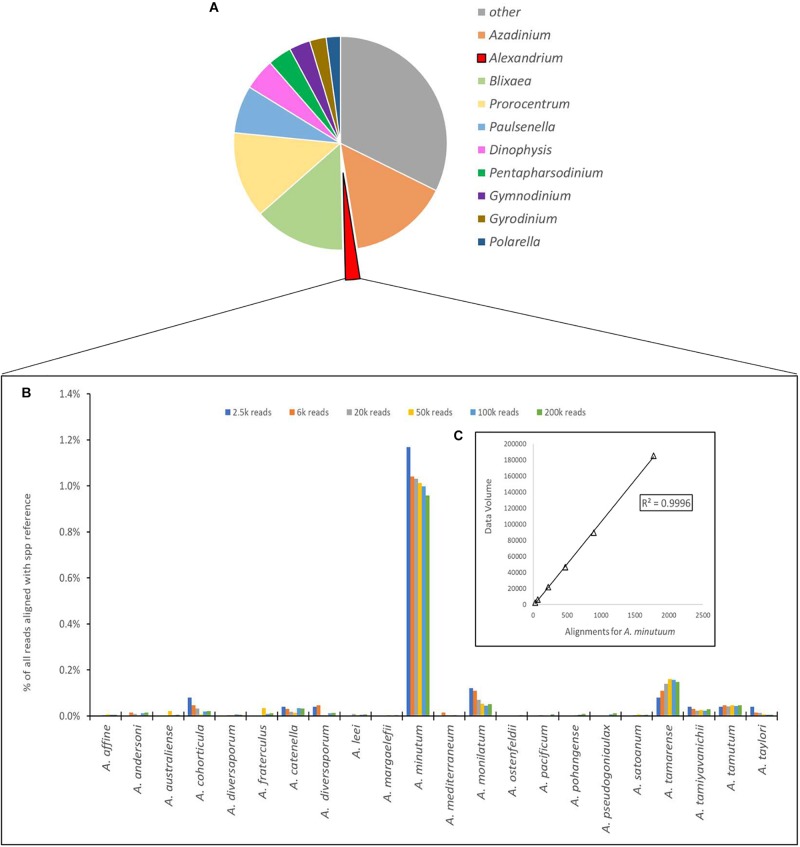
**(A)** Proportional representation of the number of sequences aligning to the 10 most common dinoflagellate genera. **(B)** Depiction of number of sequences aligned to each species of Alexandrium as a percentage of total reads for the Site 1 environmental sample, with different column colors represents volume of data used for analysis with an approximate log2 between each dataset used. **(C)** Chi-squared distribution of the *A. minutum* data throughout the changes in data volume.

The un-spiked data shown in Figure 3B was derived from a dataset containing approximately 700k reads. All samples analyzed had a low number of reads aligning with *Alexandrium* tamarense, including the negative control, indicating the potential of false positives due to contamination or “cross talk” between barcodes (Wick et al., 2018; Xu et al., 2018).

There was however, a higher prevalence of sequences aligning with *Alexandrium minutum* in Site 1, with a 95.5% average identity. Low abundances of other species were also identified, however, these had lower average identities, ranging between 83 and 88%. On investigation it was discovered that shellfish sampled from site 1 a week before the water sample was collected had low levels gonyautoxin 2&3 (GTX2 and 3), the chromatogram of which is shown in Figure 3C.

The number of sequences from the site 1 environmental sample that aligned using the EPI2ME custom alignment tool to each genus in the reference list is shown in Figure 4A. Figure 4B shows the alignments to each *Alexandrium* spp as, is shown in 4 with each column color representing a different volume of data used for analysis with an approximate log2 between each dataset. The columns associated with *Alexandrium minutum* on this graph represent the single peak for *Alexandrium minutum* in Figure 3B. Chi-squared distribution analysis of the varying volumes of data against the number of sequences aligning to *A. minutum* resulted in an *r*^2^-value of 0.9996 (Figure 4B). This is therefore indicating a small dataset is highly representative of a much larger one, however, a notably large shift in percentage was observed between the lowest two dataset volumes.

### Environmental Samples

**FIGURE 5:**
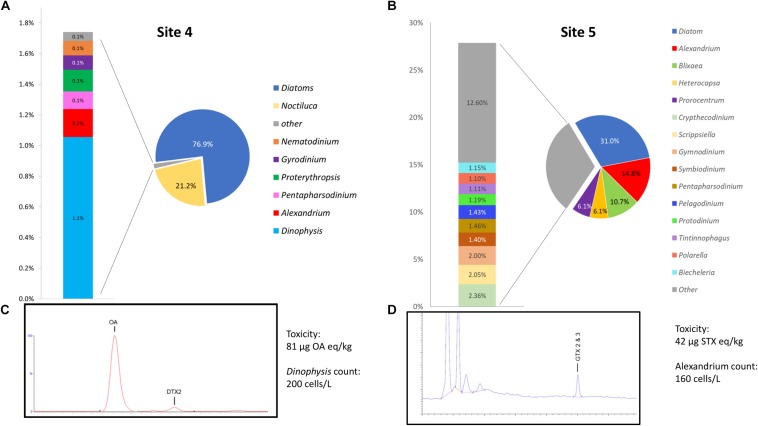
**(A,B)** Provide graphical representation of data generated by the EPI2ME custom alignment tool for environmental sites 4 and 5, cell count and toxin levels are also quoted. **(C,D)** Show respective chromatograms providing toxin profile for each site.

A graphical representation of data generated by the EPI2ME custom alignment tools analysis of environmental sites 4 and 5 can be seen in Figures 5A,B respectively. Due to the high number of species detected the figure shows only specificity to genus level, 5c and 5d show respective chromatograms providing toxin profile for each site and total toxicity and cell counts quoted next to each chromatogram.

Site 4, that was associated with a *Dinophysis* bloom event had 1.1% of the total sequences aligned to the genera, this represented the second most common alignment with a dinoflagellate. There was a notable high prevalence of diatom sequences being present (∼76%) as well as *Noctiluca* (21.1%). The distribution of *Dinophysis* sequences in site 4 was notably diverse with 53% being attributed to *D. norvegica*, 14% to both *D. acuminata* and *D. caudata*, 10% to *D. acuta*, 7% to *D. fortii*, and 2% to *D. infundibulus*. A consensus sequence generated from 20 reads that aligned to *D. norvegica* was found to be more similar to *D. acuminata* (99.59% ident with AB073117) than *D. norvegica* (99.36% ident with AB073119) when BLASTn was performed on the NCBI database.

Site 5 had 14.8% of all sequences align with *Alexandrium*, 96% with *A. minutum*, having an average identity of 95.8%. A consensus sequence generated from 20 of the *A. minutum* reads aligned best with *A. minutum* strain CCMP113 (accession JF521634.1) with BLASTn of the NCBI database (99.21% identity). As with sample 4, a significant proportion of the sequences aligned with the diatom reference sequence (∼31%).

### Method Reproducibility

The assessment of coefficient of variation highlighted that relative standard deviation (RSD) was consistently <20% with species that had >100 alignments, and RSD was consistently <35% for species that had >50 alignments (see Supplementary Material 3).

Both inter-batch reproducibility studies both found no significant differences between data sets generated by either repeat PCR preparations of the same sample or analysis of the same sample on different instruments/flow cells. The PCR inter-batch reproducibility having a *X*^2^ = 0.347 and *p* = 0.840. For the instrumental inter-batch reproducibility *X*^2^ = 1.3602 and *p* = 0.999. The within batch reproducibility ANOVA test also highlighted no significant difference between samples run simultaneously, producing a *P*-value of 0.968 with.

### Measurement of Intraspecific Variation

**FIGURE 6:**
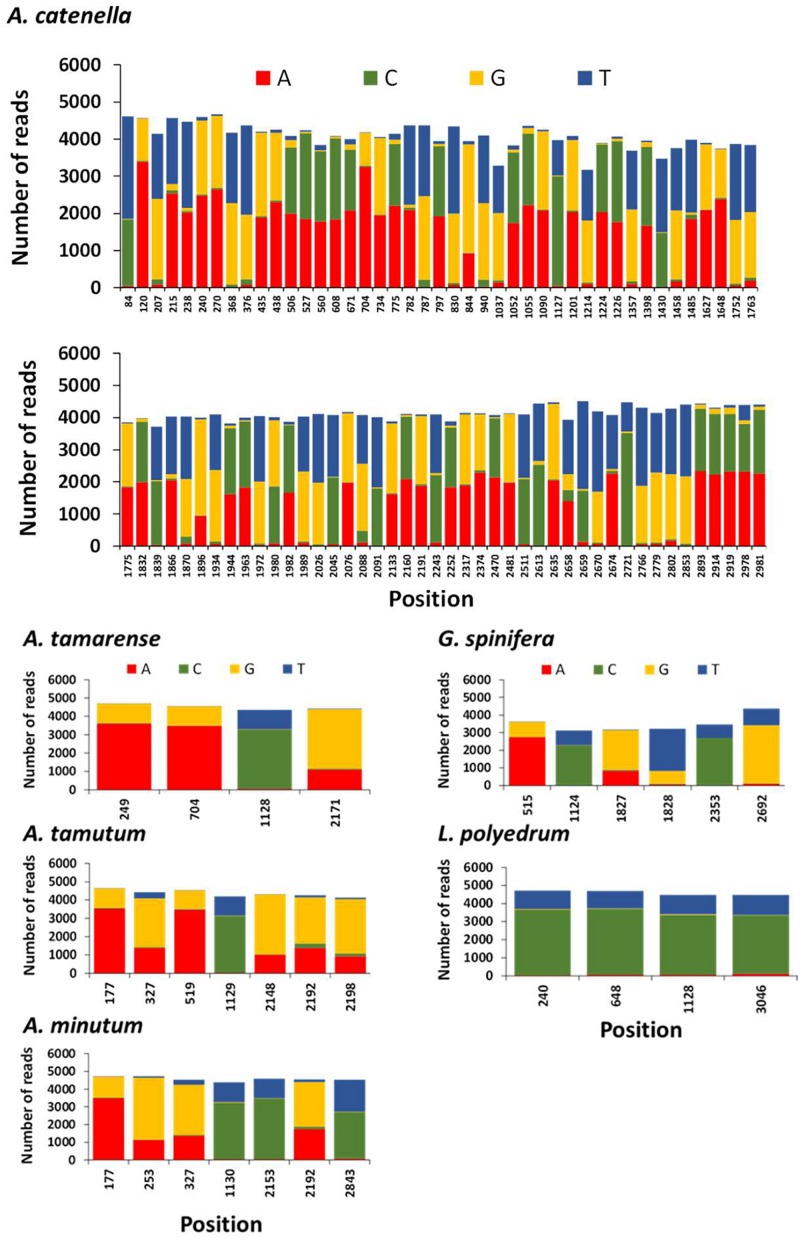
Frequency of nucleotides and position of candidate single nucleotide polymorphisms (SNPs) in each reference species using NanoPipe. A total of 5000 randomly extracted reads were used as the query and the consensus was used as the target.

The position and frequency of nucleotides of candidate single nucleotide polymorphisms (SNPs) identified in *A. tamarense, A. tamutum, A. minutum, G. spinifera*, and *L. polyedrum* are shown in Figure 6. The number of SNPs for these species ranged between 4 and 7, and the consensus used as target ranged in length between 2960 and 3022 bp. The estimated intraspecific/intragenomic variability for *A. tamarense, A. tamutum, A. minutum, G. spinifera*, and *L. polyedrum* were 0.13, 0.23, 0.23, 0.20, and 0.13%, respectively. The substitutions observed were all transitions with exception of 2 transversions observed in *G. spinifera* (Figure 6). For *A. catenella* 87 SNPs were identified with 31 transitions and 56 transversions (Figure 6), and an estimated intraspecific/intragenomic variability of 2.89%.

### qPCR Analysis

The standard curve used for quantitation produced an *r*^2^-value of 0.993 and had consistent sensitivity of 10 copies per reaction. Environmental sites 1 and 5 were both found by qPCR to be positive for the presence of *Alexandrium* cells, with average ct values of 30.48 and 23.58 respectively, equating to approximately 13 cells/L for site 1 and 321 cells/L for Site 5. All other samples were found to be negative for *Alexandrium* DNA.

## Discussion

This study examines, for the first time, the applicability of nanopore sequencing for the detection of marine eukaryotic HAB species. To achieve this, a novel method was developed, using nanopore sequencing to analyze a ∼3KB amplicon that encompassed multiple regions of the rDNA cassette. Regions, widely accepted as containing barcode genes for the speciation of dinoflagellates (John et al., 2003; Litaker et al., 2007; Orr et al., 2011; Guo et al., 2015). By performing a partial validation, using multiple matrices and a variety of reference species, both genetically similar and diverse, the robustness of the assay was examined. Comparison of an “off the shelf” data analysis tool provided by the ONT EPI2ME platform and a custom data processing pipeline highlighted that both are suitable for the discrimination of taxonomically similar organisms. The benefit of using an offline data analysis tool makes the assay suitable for field applications, however, in this instance it came at a cost of lower alignment accuracy and as such the tool would have to be developed further before being deemed fit for purpose. Conversely, the use of an “off the shelf” tool also highlights the applicability of the assay to users without bioinformatic expertise. The outputs of this study represent a valuable and a crucial first step toward a refined assay for detection of HAB species and potential utility in future wider marine monitoring. Both data analysis strategies used in this study were reliant on the curation of a custom reference list, a key part of assay development, which in this instance made use of the 18S dinoflagellate database DinoREF (Mordret et al., 2018). The selected, aligned and trimmed sequences in the reference list, only included sections of the 18S region of the rDNA cassette. As such, it did not make full use of the long-read capabilities of the technique or the more divergent ITS regions. In-spite of this, the assay performed well, with the EPI2ME data analysis consistently identifying over 99% of the pure culture sequences correctly and all consensus reads having over 99.6% alignment identity with reference sequences from the GenBank database.

The generation of consensus sequences highlighted SNPs density for *A. tamarense, A. tamutum, A. minutum, G. spinifera*, and *L. polyedrum* were considerably lower than for *A. catenella*. These results agree with the intragenomic variability reported for *A. catenella* and *A. tamarense* in SSU rDNA by Miranda et al. (2012). Miranda and her colleagues observed that the number of intragenomic SSU rDNA polymorphic sites (IRP) in “*A. catenella*” strains ranged between 0 and 50 whereas in “*A. tamarense*” strains, none or only one IRP was observed. There have been major challenges in standardizing sequence identity thresholds in order to delineate specific taxonomic groups (e.g., genus, species) in large scale eDNA datasets using HTS. These findings are important in helping to understand intra-specific diversity variation and establish thresholds for taxonomic assignment specially to discriminate complex lineages (e.g., cryptic species or closely related species). Notwithstanding, the high number of SNPs in *A. catenella* are the probable cause that both MinION and Sanger sequencing aligned well with GenBank sequences but not with each other.

The use of a mock community validated this study since we were able to identify all reference dinoflagellate species even when combined with complex environmental samples. The long-read nanopore dataset showed a relatively proportional read number between all sample sites, including within the mock community. Furthermore, by comparing the outputs of varying volumes of data from this experiment it was possible to show that relatively small datasets of tens of thousands of reads were representative of much larger datasets consisting of multiple millions of reads depending on sample complexity and requirements for downstream data processing. The contamination of the negative control material from this study highlighted the possibility of cross sample contamination, however, this could also be attributable to cross talk between barcodes. This can occur either due to barcode switching, the presence of chimeric DNA strands, or due to errors in demultiplexing with estimated prevalence being between 0.3 and 0.056% depending on study (Wick et al., 2018). The level of *A. tamarense* sequences observed in the negative control was 0.004% of the total number of reads assigned to *A. tamarense* within the library preparation run. This is in alignment with published estimations and therefore has implications on data interpretation when samples are barcoded together for throughput purposes, especially when a high prevalence of a sequence within the same batch is observed.

The identification of *Alexandrium minutum* in the un-spiked site 1 and site 5 environmental samples were both highly significant. Site 1 was initially thought to be negative for both cells and toxicity however, after nanopore sequencing identified the potential presence of *Alexandrium minutum*, a review of routine monitoring of shellfish toxin chromatograms was undertaken. This identified a low-level occurrence of Gonyautoxin 2 and 3 (GTX2 and 3) in shellfish flesh from the site at levels below the method reporting limit and therefore not present, in official documentation. Toxicity levels in site 5 at the time of sampling were below reporting limit but cell counts had breached the action limit. The high prevalence of *Alexandrium minutum* DNA in the sample when analyzed by nanopore sequencing both corroborated the cell count and the chemo-taxonomic profile of *A. minutum* (Turner et al., 2014; Lewis et al., 2018). The findings were further corroborated by qPCR analysis which also provided indication of the levels of *Alexandrium* in sites 1 and 5. The detection and characterization at sub action or reporting limit highlights for both phytoplankton cell counts, and shellfish flesh toxicity highlight potential applicability to HAB monitoring for *Alexandrium* species.

The analysis of the site 4 sample provided some indication of the assay’s performance on a species that was not characterized in the pure culture analysis, namely *Dinophysis*. The findings of which were harder to interpret than for the sites experiencing *Alexandrium* blooms. The number of sequences present as a percent of total for the sample were considerably lower for the genera (∼1.1%), with a much higher presence of diatom DNA and no single species of *Dinophysis* was identified as the clear causative organism. Furthermore, chemo-taxanomy could not definitively identify the causative organism due to the presence of both Okadaic Acid (OA) and Dinophysis toxin 2 (DTX2) in an ambiguous ratio (Dhanji-Rapkova et al., 2019). These observations are potentially explained by *Dinophysis* both correlating with diatom blooms and often not being the dominant species during a bloom (Escalera et al., 2006). A similarity matrix generated from the reference list used highlighted that there was between 98 and 99.9% similarity between the sequences. The high similarity of the 18S gene between strains of *Dinophysis* could explain the lack of clear species identification and highlights the requirement to make use of other parts of the amplicon that may be more divergent between species within the genus.

The low cost nature of nanopore sequencing will provide a platform for mass sequencing of reference cultures and environmental samples of both HAB species as well as benign phytoplankton, helping to generate better reference list(s) that will make full use of the long read nature of the technology. In doing so more accurate determination of species in environmental samples will be achieved. Furthermore, the development and application of primers to be more selective of target organisms and respective barcode regions will provide the opportunity to enhance method performance and applicability.

Sample throughput of the assay in this study was far from optimized. It is, however, envisaged that preparation of a batch of samples can be achieved in a single 8–9 h day. If this is achieved, sequencing can be performed overnight and depending on data generation rates and requirements, could be ready for data analysis the next morning. The amount of data required will be a direct result of the number of samples being run concurrently and the complexity of samples, i.e., the number of input species in each sample, with pure cultures requiring far less data than environmental samples. Overnight sequencing yield will dependent on the type of flow cell used, the low cost Flongle generates less data but also at a much slower rate, it is however possible to load a single sample on two Flongles and run them in parallel to keep costs down while ensuring enough data is generated. Experience gained from this project indicates that approximately 150,000 reads of ∼3 kb long can be generated in the 15 h between working days on a Flongle, compared 1.6 million on a regular flow cell. The time required for data analysis is dependent on computer power if being performed locally and both load on virtual machine and internet speed if being performed remotely and the volume of data being analyzed will affect both approaches. EPI2ME analysis time took approximately 50 min to align 200,000 to the 233 species in the reference list, however, data analysis times were notably variable.

Streamlining of sample preparation could be significantly aided by using the VolTRAX V2. This device manufactured by ONT, allows sample preparation it to be performed outside of laboratory environments, can multiplex samples together and reduce preparation time. The use of this hardware has allowed sequencing to be performed in a range of hostile environments (Castro-Wallace et al., 2017; Pomerantz et al., 2018).

The financial cost associated with the method are primarily dependent on the volume of data required and the resulting sequencing strategy employed. As single flow cells cost approximately $900 (USD) if purchased individually, a significant saving can be made by using disposable “Flongle” flow cells that cost about $120. The ability to re-use flow cells and multiplex more samples on to them can make the use of Flongles a false economy unless only a small amount of data is required. Multiplexing samples offers the greatest reduction in cost and allows up to 96 samples to be analyzed concurrently. Without multiplexing, the cost for a single run on a regular flow cell is very high >$1000 (USD). Multiplexing kits that are compatible with the ligations sequencing protocol allow up to 96 samples to be barcoded, depending on which kit is used (EXP-PBC001or EXP-PBC096). Once the cost of kits and additional reagents are accounted for, the cost per sample for 96 samples to be analyzed on a full flow cell equates to approximately $20–30 not including DNA extraction. This should provide >30,000 reads per barcode over a 36 h run. The flow cell could be re-used to further save money, but data generation rates and average Q scores will be lower in any subsequent experiments.

This manuscript highlights, the suitability of nanopore sequencing to the study of HAB species which have a highly significant impact on food safety, animal and ecosystem health. The perceived inaccuracy of the technology did not prevent the accurate identification of multiple species in complex environmental samples. The curation of a custom reference list and the adoption of higher accuracy and cheaper flow cells (Eisenstein, 2019; Grädel et al., 2019) will help to fulfill the potential of this exciting technology to this important area of research.

## Data Availability Statement

The datasets presented in this study can be found in online repositories. The names of the repository/repositories and accession number(s) can be found in the article/Supplementary Material. Specific datasets presented in this study can be provided on request to the corresponding author.

## Author Contributions

RH undertook the primary practical and lab work, sample collection, data analysis and manuscript production. TB contributed to concept design, specialist consultation, data analysis and manuscript review. VF was responsible for the specialist consultation, method design and manuscript production. AS analyzed the data (developed in-house data pipeline). AT and JM-U contributed to the specialist consultation, manuscript review and general guidance. AL undertook culturing of algae, specialist consultation and manuscript review. FB provided guidance for lab work, data analysis and manuscript production. KD performed the toxin analysis of shellfish samples.

## Conflict of Interest

The authors declare that the research was conducted in the absence of any commercial or financial relationships that could be construed as a potential conflict of interest.

## Footnotes

1 http://bioinformatics.uni-muenster.de/tools/nanopipe2

2 https://github.com/rrwick/Porechop

3 https://github.com/nanoporetech/qcat

4 https://github.com/fbreitwieser/pavian
