# Supplementary material for: The Application of Nanopore Sequencing Technology to the Study of Dinoflagellates: A Proof of Concept Study for Rapid Sequence-Based Discrimination of Potentially Harmful Algae
Source: Front Microbiol. 2020 May 8;11:844. doi: 10.3389/fmicb.2020.00844 (PMC7227484; doi:10.3389/fmicb.2020.00844)

# Supplementary material

## 1. Reference list used for Epi2me analysis

| Genera | Species and Accession number |
|---|---|
| Adenoides | Adenoides_eludens_AF274249.1 |
| Aduncusdinium | Aduncusdinium_glandulum_LK934662.8 |
| Ailadinium | Ailadinium_reticulatum_KJ187034.1 |
| Akashiwo | Akashiwo_sanguinea_AY831410.1 |
| Alexandrium | Alexandrium_affine_AB088282.1 |
| | Alexandrium_andersoni_JF521619.1 |
| | Alexandrium_australiense_Group_V_KF908802.1 |
| | Alexandrium_cohorticula_AF113935.1 |
| | Alexandrium_diversaporum_KF251139.1 |
| | Alexandrium_fraterculus_AY421776.1 |
| | Alexandrium_fundyense_Group_I_JF521624.1 |
| | Alexandrium_hiranoi_AY641564.1 |
| | Alexandrium_leei_AY641565.1 |
| | Alexandrium_margaelefii_U27498.1 |
| | Alexandrium_mediterraneum_group_II_KF908797.1 |
| | Alexandrium_minutum_AY831408.1 |
| | Alexandrium_monilatum_AY883005.1 |
| | Alexandrium_ostenfeldii_AJ535383.1 |
| | Alexandrium_pacificum_GroupIV_KF908800.1 |
| | Alexandrium_pohangense_LN811348.1 |
| | Alexandrium_pseudogoniaulax_AB088302.1 |
| | Alexandrium_pseudogoniaulax_JF521638.1 |
| | Alexandrium_satoanum_AY641566.1 |
| | Alexandrium_tamarense_species_group_III_KF646525.1 |
| | Alexandrium_tamiyavanichii_AB088318.1 |
| | Alexandrium_tamutum_AJ535376.1 |
| | Alexandrium_taylori_AJ535385.1 |
| Amphidiniopsis | Amphidiniopsis_rotundata_AB639343.1 |
| Amphidinium | Amphidinium_gibbosum_AB863027.1 |
| | Amphidinium_herdmanii_AF274253.1 |
| | Amphidinium_longum_AF274254.1 |
| | Amphidinium_massartii_HF674441.2 |
| | Amphidinium_mootonorum_GU295202.1 |
| | Amphidinium_operculatum_AB704006.1 |
| | Amphidinium_sp_FR865623.1 |
| Amphidoma | Amphidoma_languida_JN615412.1 |
| Amylax | Amylax_buxus_AB375868.1 |
| Amyloodinium | Amyloodinium_ocellatum_AF080096.1 |
| Ankistrodinium | Ankistrodinium_armigerum_AB858349.1 |
| Apicoporus | Apicoporus_parvidiaboli_EU293236.1 |
| Apocalathium | Apocalathium_malmogiense_KF751923.1 |
| Archaeperidinium | Archaeperidinium_minutum_AB564308.1 |
| Azadinium | Azadinium_cuneatum_KJ481822.1 |
| | Azadinium_dalianense_KF543360.1 |
| | Azadinium_obesum_GQ914935.1 |
| | Azadinium_polongum_JX559886.1 |
| | Azadinium_poporum_HQ324897.1 |
| | Azadinium_spinosum_FJ217814.1 |
| | Azadinium_trinitatum_KJ481803.1 |
| Biecheleria | Biecheleria_brevisulcata_LC068842.1 |
| | Biecheleria_cincta_FR690459.1 |
| Bispinodinium | Bispinodinium_angelaceum_AB762397.1 |
| | Blastodinium_contortum_JX473667.1 |
| | Blastodinium_crassum_FJ228702.2 |
| | Blastodinium_galatheanum_FJ541188.1 |
| | Blastodinium_mangini_JX473656.1 |
| | Blastodinium_spinulosum_HQ226071.1 |
| | Blastodinium_spinulosum_JX473663.1 |
| Blixaea | Blixaea_quinquecorne_AB246744.1 |
| Brachidinium | Brachidinium_capitatum_HM066998.1 |
| Bysmatrum | Bysmatrum_subsalsum_HQ845326.1 |
| Ceratocorys | Ceratocorys_horrida_AF022154.1 |
| | Ceratocorys_horrida_DQ388456.1 |
| Ceratoperidinium | Ceratoperidinium_falcatum_KP790150.1 |
| Chytriodinium | Chytriodinium_Atlantic_KM245128.1 |
| Cochlodinium | Cochlodinium_fulvescens_AB288380.1 |
| Coolia | Coolia_malayensis_HQ897279.1 |
| | Coolia_monotis_FR847220.1 |
| Crypthecodinium | Crypthecodinium_cohnii_FJ821501.1 |
| Cryptoperidiniopsis | Cryptoperidiniopsis_brodyi_DQ991372.1 |
| Dinophysis | Dinophysis_acuminata_FJ869120.1 |
| | Dinophysis_acuta_AJ506973.1 |
| | Dinophysis_caudata_EU780644.1 |
| | Dinophysis_fortii_AB073118.1 |
| | Dinophysis_infundibulus_AB366002.1 |
| | Dinophysis_norvegica_AJ506974.1 |
| Diplopsalopsis | Diplopsalopsis_bomba_AB261513.1 |
| Duboscquodinium | Duboscquodinium_collinii_HM483398.1 |
| Durinskia | Durinskia_agilis_JF514515.1 |

| Genera | Species and Accession number |
|---|---|
| Ensiculifera | Ensiculifera_imariensis_KR362906.1 |
| | Ensiculifera_loeblichii_HQ845328.1 |
| Fragilidium | Fragilidium_mexicanum_FJ405355.1 |
| | Fragilidium_subglobosum_AF033869.1 |
| Galeidinium | Galeidinium_rugatum_AB195668.1 |
| Gambierdiscus | Gambierdiscus_australes_AB764306.1 |
| | Gambierdiscus_caribaeus_EF202983.1 |
| | Gambierdiscus_carpenteri_KM272970.1 |
| | Gambierdiscus_pacificus_EF202872.1 |
| | Gambierdiscus_polynesiensis_EF202905.1 |
| | Gambierdiscus_ruetzleri_EF202853.1 |
| | Gambierdiscus_yasumotoi_AB764309.1 |
| Gonyaulax | Gonyaulax_cochlea_DQ388465.1 |
| | Gonyaulax_ellegaardiae_LC036590.1 |
| | Gonyaulax_fragilis_AY672702.1 |
| | Gonyaulax_polygramma_AJ833631.1 |
| | Gonyaulax_spinifera_AF022155.1 |
| | Gonyaulax_spinifera_EU805590.1 |
| | Gonyaulax_verior_AY443013.1 |
| Gotoius | Gotoius_excentricus_AB261514.1 |
| Gymnodinium | Gymnodinium_aureolum_KJ481834.1 |
| | Gymnodinium_catenatum_DQ785883.1 |
| | Gymnodinium_dorsalisulcum_DQ837534.1 |
| | Gymnodinium_galatheanum_AF272045.1 |
| | Gymnodinium_litoralis_KP790152.1 |
| | Gymnodinium_microreticulatum_AB265964.1 |
| | Gymnodinium_mikimoto_AF022195.1 |
| | Gymnodinium_nolleri_AB265963.1 |
| Gymnoxanthella | Gymnoxanthella_radiolariae_AB920349.1 |
| Gyrodiniellum | Gyrodiniellum_shiwhaense_FR720082.1 |
| Gyrodinium | Gyrodinium_cf_KP790154.1 |
| | Gyrodinium_heterogrammum_KP790158.1 |
| | Gyrodinium_rubrum_AB120003.1 |
| Herdmania | Herdmania_litoralis_AB564298.1 |
| Heterocapsa | Heterocapsa_niei_AF274265.1 |
| | Heterocapsa_pygmaea_AF274266.1 |
| | Heterocapsa_rotundata_AF274267.1 |
| | Heterocapsa_triquetra_GU594638.1 |
| Islandinium | Islandinium_minutum_AB780843.1 |
| | Islandinium_tricingulatum_AB716916.1 |
| Karena | Karena_brevis_AF274259.1 |
| Kapelodinium | Katodinium_glaucum_KP790160.1 (kapelodinium vestufic) |
| Kryptoperidinium | Kryptoperidinium_foliaceum_AF231804.1 |
| Lepidodinium | Lepidodinium_chlorophorum_AB686253.1 |
| | Lepidodinium_viride_AB686256.1 |
| Lessardia | Lessardia_elongata_AF521100.1 |
| Levanderina | Levanderina_fissa_KP790163.1 |
| Lingulodinium | Lingulodinium_polyedrum_AF274269.1 |
| Madanidinium | Madanidinium_loirii_KF751599.1 |
| Moestrupia | Moestrupia_sp_LC025894.1 |
| Nematodinium | Nematodinium_sp_FJ947038.1 |
| Niea | Niea_acanthocysta_AB273721.1 |
| | Niea_torta_AB273724.1 |
| Noctiluca | Noctiluca_scintillans_GQ380592 |
| Nusuttodinium | Nusuttodinium_myriopyrenoides_AB921301.1 |
| | Nusuttodinium_poecilochroum_AB921304.1 |
| Oodinium | Oodinium_pouchetii_KM879217.1 |
| Ostreopsis | Ostreopsis_siamensis_KT868529.1 |
| Paragymnodinium | Paragymnodinium_shiwhaense_AM408889.1 |
| Paulsenella | Paulsenella_vonstoschii_AJ968729.1 |
| Pelagodinium | Pelagodinium_beii_U37406.1 |
| Pellucidodinium | Pellucidodinium_psammophilum_LC027031.1 |
| Pentapharsodinium | Pentapharsodinium_dalei_JX262492.1 |
| | Pentapharsodinium_sp_AF274270.1 |
| | Pentapharsodinium_tyrrhenicum_AF022201.1 |
| Pernambugia | Pernambugia_tuberosa_KR362907.1 |
| Pfiesteria | Pfiesteria_piscicida_DQ991382.1 |
| Phalacroma | Phalacroma_favus_HM853786.1 |
| | Phalacroma_rapa_EU780655.1 |
| Pheopolykrikos | Pheopolykrikos_beauchampii_DQ371295.1 |
| | Pheopolykrikos_hartmanii_AY421789.1 |
| Pileidinium | Pileidinium_ciceropse_AB211357.1 |
| Polarella | Polarella_glacialis_GQ375263.1 |
| Polykrikos | Polykrikos_herdmanae_DQ975470.1 |
| | Polykrikos_kofoidii_DQ371291.1 |
| | Polykrikos_lebourae_DQ975472.1 |
| | Polykrikos_tanit_KF806599.1 |

| Genera | Species and Accession number |
|---|---|
| Prorocentrum | Prorocentrum_belizeanum_hoffmannianum_DQ238042.1 |
| | Prorocentrum_bimaculatum_HQ890882.1 |
| | Prorocentrum_concavum_Y16237.1 |
| | Prorocentrum_emarginatum_Y16239.1 |
| | Prorocentrum_foraminosum_JX912166.1 |
| | Prorocentrum_fukuyoi_JX912167.1 |
| | Prorocentrum_glenanicum_GU327678.1 |
| | Prorocentrum_glenanicum_GU327679.1 |
| | Prorocentrum_gracile_AY443019.1 |
| | Prorocentrum_levis_DQ238043.1 |
| | Prorocentrum_lima_EF377326.1 |
| | Prorocentrum_maculosum_Y16236.1 |
| | Prorocentrum_mexicanum_Y16232.1 |
| | Prorocentrum_micans_EU780638.2 |
| | Prorocentrum_minimum_EU780639.1 |
| | Prorocentrum_panamensis_Y16233.1 |
| | Prorocentrum_pseudopanamense_GU327677.1 |
| | Prorocentrum_shikokuense_AB781324.1 |
| | Prorocentrum_texanum_JQ390504.1 |
| | Prorocentrum_tsawwassenense_EF657885.1 |
| | Prorocentrum_texanum_JQ390504.1 |
| | Prorocentrum_tsawwassenense_EF657885.1 |
| Proterythropsis | Proterythropsis_sp_FJ947036.1 |
| Protoceratium | Protoceratium_reticulatum_AB727654.1 |
| Protodinium | Protodinium_simplex_JF791031.1 |
| Protoperidinium | Protoperidinium_abei_AB181881.1 |
| | Protoperidinium_abei_AB181882.1 |
| | Protoperidinium_claudicans_AB255833.2 |
| | Protoperidinium_conicum_AB181884.1 |
| | Protoperidinium_conicum_AB181885.1 |
| | Protoperidinium_crassipes_AB181888.1 |
| | Protoperidinium_denticulatum_AB181890.1 |
| | Protoperidinium_depressum_AB255834.1 |
| | Protoperidinium_divergens_AB181892.1 |
| | Protoperidinium_elegans_AB255835.1 |
| | Protoperidinium_excentricum_AY443021.1 |
| | Protoperidinium_fukuyoi_AB780842.1 |
| | Protoperidinium_leonis_AB181898.1 |
| | Protoperidinium_pallidum_AB261516.1 |
| | Protoperidinium_pellucidum_AY443022.1 |
| | Protoperidinium_pentagonum_AB255837.1 |
| | Protoperidinium_punctulatum_AB261517.1 |
| | Protoperidinium_thulesense_AB261519.1 |
| Pseudadenoides | Pseudadenoides_kofoidii_LC002843.1 |
| Pseudopfiesteria | Pseudopfiesteria_shumwayae_AF080098.1 |
| Pyrocystis | Pyrocystis_lunula_AF274274.1 |
| Pyrocystis | Pyrocystis_noctiluca_AF022156.1 |
| Pyrodinium | Pyrodinium_bahamense_AB936753.1 |
| | Pyrodinium_bahamense_AF274275.1 |
| Pyrophacus | Pyrophacus_steinii_AY443024.1 |
| Qia | Qia_lebouriae_AB261512.1 |
| Roscoffia | Roscoffia_capitata_AF521101.1 |
| Sabulodinium | Sabulodinium_undulatum_DQ975473.1 |
| Scrippsiella | Scrippsiella_acuminata_HQ845330.1 |
| | Scrippsiella_sweeneyae_HQ845331.1 |
| Sinophysis | Sinophysis_ebriola_JQ996372.1 |
| | Sinophysis_grandis_JQ996375.1 |
| Spiniferodinium | Spiniferodinium_galeiforme_AB921297.1 |
| Stoeckeria | Stoeckeria_algicida_AJ841809.1 |
| | Stoeckeria_sp_FN557541.1 |
| Symbiodinium | Symbiodinium_clade_D_DQ838542.1 |
| | Symbiodinium_goreaui_EF036539.1 |
| | Symbiodinium_microadriaticum_M88521.1 |
| | Symbiodinium_sp_AB016539.1 |
| | Symbiodinium_sp_B_KC848880.1 |
| | Symbiodinium_sp_Clade_E_AF238263.1 |
| | Symbiodinium_sp_clade_A_KU188515.1 |
| | Symbiodinium_sp_clade_C_EF419291.1 |
| Testudodinium | Testudodinium_corrugatum_AB704003.1 |
| | Testudodinium_testudo_AB704002.1 |
| Thecadinium | Thecadinium_kofoidii_AY238478.1 |
| Thoracosphaera | Thoracosphaera_heimii_AF274278.1 |
| Tintinnophagus | Tintinnophagus_acutus_HM483397.1 |
| Torodinium | Torodinium_robustum_KP790166.1 |
| Warnowia | Warnowia_sp_KP790169.1 |
| Zooxanthella | Zooxanthella_Scrippsiella_nutricula_U52357.1 |
| **Diatom** | Leptocylindrus_danicus_KC814808 |
| | Stephanodiscus_hantzschii_GQ844874 |

2. Alignments between Sanger and MinION consensus reads with differences highlighted in red.

```
Sanger
||||||
MinION
```

*L. polyedrum*:

```
CATTCGCACAGGCTTTGTGGATCAGTATGTGTTCTCATCGGGATGTTGGTCTCTGTGCTG   60
||||||||||||||||||||||||||||||||||||||||||||||||||||||||||||
CATTCGCACAGGCTTTGTGGATCAGTATGTGTTCTCATCGGGATGTTGGTCTCTGTGCTG   1838

CTCAGGCTGACTGCTAATGATGCAGTGCAATGACCTGCGGGCGACCATGCAGGCAGGCTT   120
||||||||||||||||||||||||||||||||||||||||||||||||||||||||||||
CTCAGGCTGACTGCTAATGATGCAGTGCAATGACCTGCGGGCGACCATGCAGGCAGGCTT   1898

TTCCGTAAAATCTGCCGTGGTTGCTGGTCTTGTGCCTGATGCTTAACAAATTGTTTTTAT   180
||||||||||||||||||||||||||||||||||||||||||||||||||||| ||||||
TTCCGTAAAATCTGCCGTGGTTGCTGGTCTTGTGCCTGATGCTTAACAAATTG-TTTTAT   1957

GTGAATGCACGTGCTCGTGTTATTCAGTAATGGATGCCTTGGTCTGAAAATCGATGAAGA   240
||||||||||||||||||||||||||||||||||||||||||||||||||||||||||||
GTGAATGCACGTGCTCGTGTTATTCAGTAATGGATGCCTTGGTCTGAAAATCGATGAAGA   2017

GTACAGCGAAATGTGATATGCATTGTGAACTGCAGACTTCCGTGAGTCAATGGCTTGTTG   300
||||||||||||||||||||||||||||||||||||||||||||||||||||||||||||
GTACAGCGAAATGTGATATGCATTGTGAACTGCAGACTTCCGTGAGTCAATGGCTTGTTG   2077

AACGTTTGCTGTGCCTTTGGGATACGCCTGAAGGCGCGCCTGCTTCAGCGTCTGTTCCAG   360
||||||||||||||||||||||||||||||||||||||||||||||||||||||||||||
AACGTTTGCTGTGCCTTTGGGATACGCCTGAAGGCGCGCCTGCTTCAGCGTCTGTTCCAG   2137

CATGTGCATGCCGTGCAACTGTCTTGCTTTAAGTGGCAGCGGTACTGTGGGCCTTTGCAT   420
||||||||||||||||||||||||||||||||||||||||||||||||||||||||||||
CATGTGCATGCCGTGCAACTGTCTTGCTTTAAGTGGCAGCGGTACTGTGGGCCTTTGCAT   2197
```

```
GTTACAGTGCTAACGTGTAGAGTTCAATGCGAACTCGTTGGCGAGCATTTTGTGGGACAG  480
||||||||||||||||||||||||||||||||||||||||||||||||||||||||||||
GTTACAGTGCTAACGTGTAGAGTTCAATGCGAACTCGTTGGCGAGCATTTTGTGGGACAG  2257

GAGGTGCTTAAGCGAGTGCTTTGCTAGCGCGAAGAAAGCAACGTGCATTTGCGGACTTTG  540
||||||||||||||||||||||||||||||||||||||||||||||||||||||||||||
GAGGTGCTTAAGCGAGTGCTTTGCTAGCGCGAAGAAAGCAACGTGCATTTGCGGACTTTG  2317

GAAGACATGAAGCTAGGCGAGTCAACCTGCTGAATTTAAG
||||||||||||||||||||||||||||||||||||||||
GAAGACATGAAGCTAGGCGAGTCAACCTGCTGAATTTAAG
```

*G. spinifera*

```
TTTCTGGCTCAGGCTATCATCTGTATGTTAGTCTTGTGTGCCAAAGGGCAATTGCCTGTC  60
||||||||||||||||||||||||||||||||||||||||||||||||||||||||||||
TTTCTGGCTCAGGCTATCATCTGTATGTTAGTCTTGTGTGCCAAAGGGCAATTGCCTGTC  61

CTGTGAGGACTTGCTCTTGCCAGGTTGCAAATGTTGTACGCTGACTGTGCTCTCACTTGT  120
||||||||||||||||||||||||||||||||||||||||||||||||||||||||||||
CTGTGAGGACTTGCTCTTGCCAGGTTGCAAATGTTGTACGCTGACTGTGCTCTCACTTGT  121

GCAGCAAATCATTTTCAACTATGGATGCGCTTAATTCAAACAACGATGAAGGGTGCAGTG  180
|||||||||||||||||||||||||||| |||||||||||||||||||||||||||||||
GCAGCAAATCATTTTCAACTATGGATGC-CTTAATTCAAACAACGATGAAGGGTGCAGTG  180

AAGTGTGCTAATCATTGTGACTTGCAGAATTCCGTGAACCAATGGGTTGTTGAATGTGTC  240
||||||||||||||||||||||||||||||||||||||||||||||||||||||||||||
AAGTGTGCTAATCATTGTGACTTGCAGAATTCCGTGAACCAATGGGTTGTTGAATGTGTC  240

ATGTGCTTTGGGATTCTTCCTGAAAGCATGCCTTCTTCAGAGTTTTTGCTTTGTGCTGCA  300
||||||||||||||||||||||||||||||||||||||||||||||||||||||||||||
ATGTGCTTTGGGATTCTTCCTGAAAGCATGCCTTCTTCAGAGTTTTTGCTTTGTGCTGCA  300

AATGCATGTCGTTTGCTTTGCTTTTGGGATGGCAATAATCATTGCTGTCTGCCTCTCAAG  360
||||||||||||||||||||||||||||||||||||||||||||||||||||||||||||
AATGCATGTCGTTTGCTTTGCTTTTGGGATGGCAATAATCATTGCTGTCTGCCTCTCAAG  360
```

```
GGTTGCTTGCAGACTGTATGCTAATCGCAAAGGTTGTGCAATCTTTGTCCTTGCTTCTGT   420
||||||||||||||||||||||||||||||||||||||||||||||||||||||||||||
GGTTGCTTGCAGACTGTATGCTAATCGCAAAGGTTGTGCAATCTTTGTCCTTGCTTCTGT   420

GGTGTGCCCTCTGGGTTTCCACTGGCTGAAAACATGATGTTAGGCAAGCAGACCTGCTGA   480
||||||||||||||||||||||||||||||||||||||||||||||||||||||||||||
GGTGTGCCCTCTGGGTTTCCACTGGCTGAAAACATGATGTTAGGCAAGCAGACCTGCTGA   480

ATTTAA
||||||
ATTTAA
```

*A. catenella*

```
TGTGTGACTTGTTTTGCAATCATGCGTTATGCATTGCATAAACAAGTTTGGTAAACTGAG   60
||||||||||||||||||| ||| ||||||||||||||||||||||||||||||||||||
TGTGTGACTTGTTTTGCAAACATTCGTTATGCATTGCATAAACAAGTTTGGTAAACTGAG   60

TGGTGCTGTGTTTGTGTACGATGATTATGTTTTGCAAAGAATGTTTTAGTTCAATAAATG   120
||||||||||||||||||||||||||| ||||||||| |||||||||||||||||| |||
TGGTGCTGTGTTTGTGTACGATGATTAGGTTTTGCAACGAATGTTTTAGTTCAATACATG   120

ATGAATAATGCAGGAAAATGCATTATGCATTGTGAATTGCAGAATTCCGTGAGCTAACAG   180
||||||||||||||| |||||| ||||||||||||||||||||||||||||||||||||
ATGAATAATGCAGGACAATGCAGTATGCATTGTGAATTGCAGAATTCCGTGAGCTAACAT   180

ATGTTTGAATGTTACTTGTGCCTTTGGGATATTCTTGAAGGTTTGCTTGATTCAATGCCA   240
|||||||||||||||||| |||||||||||||||||||||||||||||| ||||||||||
ATGTTTGAATGTTACTTGCGCCTTTGGGATATTCTTGAAGGTTTGCTTGGTTCAATGCCA   240

ATTACCTTCCATATGCAATACTGCTGTTTAGCATTGCTGTGAACAAGGAGAGTCAATGTG   300
| || |||||||||||||||||||||||||||||||||||||||||||||||||||||||
AGTATCTTCCATATGCAATACTGCTGTTTAGCATTGCTGTGAACAAGGAGAGTCAATGTG   300

TATTGCATTGAACCTGGATGTGTTGCAGCTGC-TTTGCAACCTAAACATGGTTGATTGGG   359
||||||||||||| |||||||||||||||||| |||||||||||| ||||||||||||||
TATTGCATTGAACATGGATGTGTTGCAGCTGCTTTTGCAACCTAAGCATGGTTGATTGGG   360

GGCAAACTTGTTTCGTCATGTGCTGGTTGCTGT   392
|||||||||||||||||||||||||||||||||
GGCAAACTTGTTTCGTCATGTGCTGGTTGCTGT   393
```

*A. fundyense*

```
GATATTGTGGGCGGTGTAAGCTTGCATTACAATAATATTACATGTGCCCTGGGCTGTGTG   60
||||||||||||||||||||||||||||||||||||||||||||||||||||||||||||
GATATTGTGGGCGGTGTAAGCTTGCATTACAATAATATTACATGTGCCCTGGGCTGTGTG   60
```

```
ACTTGTTTTGCAAACATTCGTTATGCATTGCATAAACAAGTTTGGTAAACTGAGTGGTGC  120
||||||||||||||||||||||||||||||||||||||||||||||||||||||||||||
ACTTGTTTTGCAAACATTCGTTATGCATTGCATAAACAAGTTTGGTAAACTGAGTGGTGC  120

TGTGTTTGTGTACGATGATTAGGTTTTGCAACGAATGTTTTAGTTCAATACATGATGAAT  180
||||||||||||||||||||||||||||||||||||||||||||||||||||||||||||
TGTGTTTGTGTACGATGATTAGGTTTTGCAACGAATGTTTTAGTTCAATACATGATGAAT  180

AATGCAGGACAATGCATTATGCATTGTGAATTGCAGAATTCCGTGAGCTAACATATGTTT  240
|||||||||||||||| |||||||||||||||||||||||||||||||||||||||||||
AATGCAGGACAATGCAGTATGCATTGTGAATTGCAGAATTCCGTGAGCTAACATATGTTT  240

GAATGTTACTTGCGCCTTTGGGATATTCTTGAAGGTTTGCTTGGTTCAATGCCAATTATC  300
||||||||||||||||||||||||||||||||||||||||||||||||||||||| ||||
GAATGTTACTTGCGCCTTTGGGATATTCTTGAAGGTTTGCTTGGTTCAATGCCAAGTATC  300

TTCCATATGCAATACTGCTGTTTAGCATTGCTGTGAACAAAGAGAGTCAATGTGTATTGC  360
|||||||||||||||||||||||||||||||||||||||| |||||||||||||||||||
TTCCATATGCAATACTGCTGTTTAGCATTGCTGTGAACAAGGAGAGTCAATGTGTATTGC  360

ATTGAACATGGATGTGTTGCAGCTGCTTT  389
|||||||||||||||||||||||||||||
ATTGAACATGGATGTGTTGCAGCTGCTTT  389
```

*A. minutum*

```
ACGACTTTGTGAGCTGTGGTGGGGTTCCTAGGCTTTAGGTTCTGCATCATTTGCTCGTGG  61
||||||||||||||||||||||||||||||||||||||||||||||||||||||||||||
ACGACTTTGTGAGCTGTGGTGGGGTTCCTAGGCTTTAGGTTCTGCATCATTTGCTCGTGG  60

GTGGCATGGCTTGCTTCTGCAAGCGCTTTCATGCTGCTGTGTTGATGACCTTTTGTTGAT  121
|||||||||||||||||||||||||||||||||||||||||||||||||| |||||||||
GTGGCATGGCTTGCTTCTGCAAGCGCTTTCATGCTGCTGTGTTGATGACC-TTTGTTGAT  119

TGCTTGTACTTGTTTCTTGCATTGAACTTGAATGTGAAATGTGTTTTTGCAATGAATGTC  181
||||||||||||||||||||||||||||||||||||||||||||||||||||||||||||
TGCTTGTACTTGTTTCTTGCATTGAACTTGAATGTGAAATGTGTTTTTGCAATGAATGTC  179

TTAGCTCAATTGATGATGAAGAATGCAGCAAAATGTGATATGCATTGTGAATTGCAGAAT  241
||||||||||||||||||||||||||||||||||||||||||||||||||||||||||||
TTAGCTCAATTGATGATGAAGAATGCAGCAAAATGTGATATGCATTGTGAATTGCAGAAT  239

TCCGTGAGCCAATAGATGTTTGAACGTAATTTGCACCTTCGGGATATGCTTGAAGGTGTG  301
||||||||||||||||||||||||||||||||||||||||||||||||||||||||||||
TCCGTGAGCCAATAGATGTTTGAACGTAATTTGCACCTTCGGGATATGCTTGAAGGTGTG  299
```

```
CTTGATTCAATGTCAATTAACTTCCAACATTGAATTTGCTGTTCAGCAACGTTGTGAGCT  361
||||||||||||||||||||||||||||||||||||||||||||||||||||||||||||
CTTGATTCAATGTCAATTAACTTCCAACATTGAATTTGCTGTTCAGCAACGTTGTGAGCT  359

GTGTGTGTCAATGCTGTTGCATTGGACACCCGCGCTTGCGAATGCATTGCAACCTCATTG  421
||||||||||||||||||||||||||||||||||||||||||||||||||||||||||||
GTGTGTGTCAATGCTGTTGCATTGGACACCCGCGCTTGCGAATGCATTGCAACCTCATTG  419

TGTTTGCTTAGGTCTAGCCTTTGTCACTTGCATTGGTTGCATAGTATTTGTCTGGGTAGC  481
||||||||||||||||||||||||||||||||||||||||||||||||||||||||||||
TGTTTGCTTAGGTCTAGCCTTTGTCACTTGCATTGGTTGCATAGTATTTGTCTGGGTAGC  479

TGAACAGCGTAAACTTAACATGAAGTGAAGCATGTAAACCTGCTGAATTTA
|||||||||||||||||||||||||||||||||||||||||||||||||||
TGAACAGCGTAAACTTAACATGAAGTGAAGCATGTAAACCTGCTGAATTTA
```

*A. tamutum*

```
AATCTTTCACGATTTTGTGAGCTGTGGGTGGTTTCCATTCGTTGGGTTCTGCTTCATTTG  61
||||||||||||||||||||||||||||||||||||||||||||||||||||||||||||
AATCTTTCACGATTTTGTGAGCTGTGGGTGGTTTCCATTCGTTGGGTTCTGCTTCATTTG  60

CTCGAGGGTGGCATGGCTTGCAATAGCAAGCGCTTTCATGCTGCTGTGTACATGAACTTC  121
||||||||||||||||||||||||||||||||||||||||||||||||||||||||||||
CTCGAGGGTGGCATGGCTTGCAATAGCAAGCGCTTTCATGCTGCTGTGTACATGAACTTC  120

AACTTCTTGGCTTCATGCTCGTTCTTTGCTTGCAATTAGAATGCGAAATGTGTTTTTGCA  181
|||||||||||||||||||||||||||||||||||||||||||||||||||| |||||||
AACTTCTTGGCTTCATGCTCGTTCTTTGCTTGCAATTAGAATGCGAAATGTG-TTTTGCA  179

ATGAATGTCTTAGCTCAATTGATGATGAAGAATGCAGCAAAATGTGATATGCATTGTGAA  241
||||||||||||||||||||||||||||||||||||||||||||||||||||||||||||
ATGAATGTCTTAGCTCAATTGATGATGAAGAATGCAGCAAAATGTGATATGCATTGTGAA  239
```

```
TTGCAGAATTCCGTGAGCCAATAGATGTTTGAACGTAATTTGCACCTTCGGGATATGCTT  301
||||||||||||||||||||||||||||||||||||||||||||||||||||||||||||
TTGCAGAATTCCGTGAGCCAATAGATGTTTGAACGTAATTTGCACCTTCGGGATATGCTT  299

GAAGGTGTGCTTGATTCAATGTCAATGACCTTCCAACACTCAGTTTGCTGTTCAGCAACG  361
||||||||||||||||||||||||||||||||||||||||||||||||||||||||||||
GAAGGTGTGCTTGATTCAATGTCAATGACCTTCCAACACTCAGTTTGCTGTTCAGCAACG  359

TTGTGAGCTGTGTGTGTCAATGCTGCTGCATTGGACACACGCGCTTGCGAACGCATTGCA  421
||||||||||||||||||||||||||||||||||||||||||||||||||||||||||||
TTGTGAGCTGTGTGTGTCAATGCTGCTGCATTGGACACACGCGCTTGCGAACGCATTGCA  419

ACCTTAACGTGTTTGCTTAGGTCTTACCTCTGTCACTTGCTTTGGTTGCATCGCATTTGT  481
||||||||||||||||||||||||||||||||||||||||||||||||||||||||||||
ACCTTAACGTGTTTGCTTAGGTCTTACCTCTGTCACTTGCTTTGGTTGCATCGCATTTGT  479

GCGTAATCTGAAAAGCGTAAACTTAACATGAAGTGAAGCATGTAAACCTGCTGAATTTAA  541
||||||||||||||||||||||||||||||||||||||||||||||||||||||||||||
GCGTAATCTGAAAAGCGTAAACTTAACATGAAGTGAAGCATGTAAACCTGCTGAATTTAA  539

GCATAT
||||||
GCATAT
```

*A. tamarense*

```
CAACTTCATTCTAATGATATTGTGGGCAAGTGCGGGCATGTATTGCAATGTGCTTGCATG  61
||||||||||||||||||||||||||||||||||||||||||||||||||||||||||||
CAACTTCATTCTAATGATATTGTGGGCAAGTGCGGGCATGTATTGCAATGTGCTTGCATG  61

TGCCCTGGGCTGCATGACTTGTTTTACAATCATGTGTGCTGCACTATCTAATATACTTAA  121
||||||||||||||||||||||||||||||||||||||||||||||||||||||||||||
TGCCCTGGGCTGCATGACTTGTTTTACAATCATGTGTGCTGCACTATCTAATATACTTAA  121
```

```
TCAACTGTTTGGTAATTCTTCATTGATTACAATGATTATGTTTTGCAAAGAATGTATTAG  181
||||||||||||||||||||||||||||||||||||||||||||||||||||||||||||
TCAACTGTTTGGTAATTCTTCATTGATTACAATGATTATGTTTTGCAAAGAATGTATTAG  181

TTCAATAAATGATGAAGAATGCAGCAAAATGCACTATGCATTGTGAATTGCAGAATTCCG  241
||||||||||||||||||||||||||||||||||||||||||||||||||||||||||||
TTCAATAAATGATGAAGAATGCAGCAAAATGCACTATGCATTGTGAATTGCAGAATTCCG  241

TGAGCTAACAGATGTTTGAATGTTACTTGTACCTTTGGGATATTCTTGAAGGTGTGCTTG  301
||||||||||||||||||||||||||||||||||||||||||||||||||||||||||||
TGAGCTAACAGATGTTTGAATGTTACTTGTACCTTTGGGATATTCTTGAAGGTGTGCTTG  301

ATTCAATGCAAACTGTCTTCCATATGCAATAATGCTGCTTAGCATTGCTGTGAACAGTAG  361
||||||||||||||||||||||||||||||||||||||||||||||||||||||||||||
ATTCAATGCAAACTGTCTTCCATATGCAATAATGCTGCTTAGCATTGCTGTGAACAGTAG  361

GGGTCAATGTGTGTGCATTGAACCTGGGTGTTGTGCAGCTGTTTGCAACCTAAACATGTT  421
||||||||||||||||||||||||||||||||||||||||||||||||||||||||||||
GGGTCAATGTGTGTGCATTGAACCTGGGTGTTGTGCAGCTGTTTGCAACCTAAACATGTT  421

TTCTTGGGGGCAAACTTGTTTCGTCATTTGCTGGTTGATATGTGTAAAAATGCATATTTG  481
||||| |||||||||||||||||||||||||||||||||||||||| |||||||||||||
TTCTT-GGGGCAAACTTGTTTCGTCATTTGCTGGTTGATATGTGT-AAAATGCATATTTG  479

AAACAAGTTGAATACTTGCATTCACTTAGCGTGAAGTGAAGCACATAAACCTGCTGAATT  541
||||||||||||||||||||||||||||||||||||||||||||||||||||||||||||
AAACAAGTTGAATACTTGCATTCACTTAGCGTGAAGTGAAGCACATAAACCTGCTGAATT  539

TAA
|||
TAA
```

3. Coefficient of variation, with percent relative standard deviation plotted against number of alignments per species.

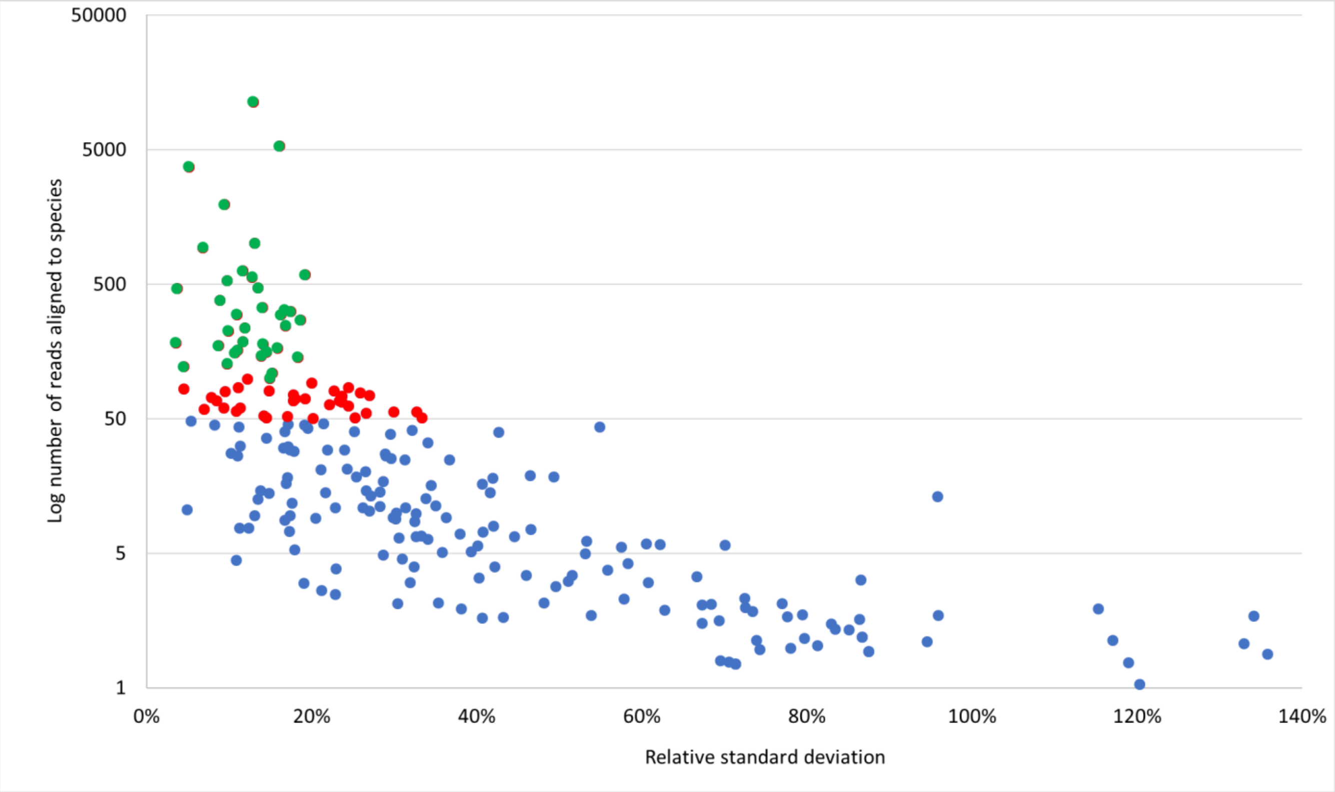

Supplement: Supplementary file 1 [file Data_Sheet_1.pdf]
